# The effect of in-person and virtual prenatal care education of the spouses of primiparous women on the father and mother’s attachment to infant: a quasi-experimental and controlled study

**DOI:** 10.1186/s13063-021-05559-0

**Published:** 2021-09-03

**Authors:** Zari Doaltabadi, Leila Amiri-Farahani

**Affiliations:** 1 Student Research Committee, Department of Reproductive Health and Midwifery, School of Nursing and Midwifery, Iran University of Medical Sciences, Tehran, Iran; 2 Department of Reproductive Health and Midwifery, Nursing Care Research Center, School of Nursing and Midwifery, Iran University of Medical Sciences, Tehran, Iran

**Keywords:** Father-infant attachment, Mother-infant attachment, Prenatal care, Social networks, Virtual education, Face-to-face education

## Abstract

**Introduction:**

Considering the important role of education in promoting parents’ attachment to the infant, temporal and spatial limitations, and the need to use new educational methods for spouses’ participation in childbirth preparation classes, the present study was conducted to compare the effect of in-person and virtual prenatal care education of the spouses of primiparous women on the father and mother’s attachment to the infant.

**Methods:**

This is a quasi-experimental clinical trial that was conducted on primiparous pregnant women referring to three prenatal clinics in Tehran, Iran. Sampling was done by continuous method and pregnant women were divided into three groups of face-to-face education (*n* = 28), virtual education (*n* = 31), and control (*n* = 29). The content of the training program in the virtual and face-to-face groups was similar, which was presented in 4 sessions. At 18–20 weeks of gestation, demographic characteristics and pregnancy records were obtained from the samples, and 12 weeks after the delivery, maternal postnatal attachment scale, and postnatal paternal-infant attachment questionnaire were completed. Both intention-to-treat analysis and per-protocol analysis were performed.

**Results:**

There was a statistically significant difference between the two groups of in-person education and control, and also virtual education and control for both intention-to-treat and per-protocol analysis (*p* < 0.05). However, no statistically significant difference was found between the two groups of in-person and virtual education. Results showed a large and medium effect size between the two groups of in-person education and control, and virtual education and control in terms of father-infant attachment score, respectively. There was also no statistically significant difference between the three groups after the educational intervention in terms of the mother-infant attachment score for both intention-to-treat and per-protocol analysis.

**Conclusion:**

Considering that education by both in-person and virtual methods had the same effect on improving the score of father-infant attachment, it is suggested that to increase the participation of spouses of pregnant women in the process of prenatal care, the spouses of pregnant women should have the option of virtual education in addition to in-person training.

**Trial registration:**

TCTR.ir TCTR20200515011. Registered on May 12, 2020.

**Supplementary Information:**

The online version contains supplementary material available at 10.1186/s13063-021-05559-0.

## Introduction

Attachment in developmental psychology refers to the emotional bond that develops between an infant and a mother or another adult. Attachment occurs when there is a warm, intimate, and lasting relationship between the infant and caregiver [1]. In pregnancy, parents begin a journey of expectancy and hope, with immense personal investment in a new future with their baby. New life is expected and experienced as pregnancy progresses, and for most couples, it is a time of great joy as bonds of attachment are formed with a new baby [2].

Parents have an emotional relationship with the fetus while they are waiting for their baby to be born [3]. The onset of this relationship occurs before birth [4] and increases with the sensation of fetal movements by the mother, reaching its peak after the birth [5]. The pattern of attachment changes over time and improves after delivery through eye contact and olfactory and tactile touch of mother and infant [6]. Creating a secure attachment in infancy enables the child to establish close relationships with parents, especially in older age [7]. In contrast, infants who have had less attachment and relationship with parents may have lower emotional and mental development, poorer social interactions, and reduced ability to communicate in the long term, along with more aggressive and hostile behaviors than their peers [8, 9]. The attachment between mother and infant plays an important role in the normal development and physical-emotional development of the infant, and the mother’s attachment style determines how the infant relates to the world around him/her [10]. Mothers who have a stronger and healthier attachment to their fetus during pregnancy interact better with their infants, which in turn can have a significant impact on the infant’s emotional, cognitive, and social development [7]. The stages of attachment in the father are somewhat similar to the mother, but the time of attachment in the father is different from the mother. Although mothers show a rapid increase in the attachment in the fifth month of pregnancy and have a special feeling during pregnancy, this feeling occurs in fathers more slowly and sharply increases after birth and during infant care [5].

Factors such as experiencing and counting fetal movements [11], listening to music [12], giving massage [13], family support [14], mother-to-child skin contact [15], kangaroo care immediately after birth [16], and prenatal education [17] have a positive effect on creating attachment.

Prenatal education is a dynamic process through which, the couples become aware of the physical and mental changes during pregnancy, acquire the skills needed to overcome the fear of childbirth, and learn supportive techniques that make them an informed parents [18]. Educating fathers along with their wives can increase the effectiveness of attachment education programs [19]. Paternal education and information about the importance of breastfeeding and infant issues significantly reduce anxiety in fathers, and fathers who participate in infant care training programs have a significantly lower stress level [20, 21]. Today, due to social changes such as increasing maternal employment and the emergence of small and isolated families, fathers are taking care of their children more than before. A great deal of evidence supports the important role of the father’s involvement in improving maternal and infant outcomes. Thus, the role of educating fathers during pregnancy becomes more prominent [22].

The results of studies on the level of participation of spouses in supporting pregnant women show that the level of participation varies in different parts of the world. In Iran, compared to developed countries, the participation of Iranian fathers in pregnancy care is low [23]. The qualitative study of Mortazavi and Mirzaei showed that factors such as lack of sufficient knowledge on pregnancy, work issues, and feminine environment of the centers that provide prenatal care are among the barriers to fathers' participation in pregnancy care [24].

By using new educational methods such as virtual education, problems such as lack of time, lack of special places for education, and the need for experienced and trained educators can be solved. Electronic learning (e-learning) can solve some of the problems that currently exist in the field of childbirth preparation classes in Iran through its interactive, self-guiding, and flexible nature that does not require specific time and space [25]. The use of different educational methods is essential for the fathers’ participation in prenatal care (24). A Men's training program is more successful if it is supported by mass media, community organizations, and public education, and meets the men’s needs [26]. Considering the abovementioned barriers to fathers' participation in prenatal care and also the ease of access to the internet and virtual mass media in Iran, the present study was conducted with the aims of (1) comparing the effect of in-person and virtual prenatal care education of the spouses of primiparous women on the mother’s attachment to the infant and (2) comparing the effect of in-person and virtual prenatal care education of the spouses of primiparous women on the father’s attachment to the infant.

## Methods

### Design

This is a quasi-experimental study with three groups of in-person training (parallel design), virtual training, and control. Reporting of this study is in accordance with the Consolidation Standards of Reporting Trials (CONSORT) statement (Additional file 1) [27]. The study population consisted of the spouses of primiparous pregnant women referred to the prenatal clinics of Lolagar Hospital (virtual education group), Azadi Town Health Center (in-person training group), and Tehransar Health Center (control group) (Table 1). Three health centers were selected from areas with similar socio-economic status in the city of Tehran. Also, all the selected centers were public centers and were providing health services free of charge. The educational content was designed according to the educational needs of men to participate in prenatal care [26]. The first session was held in week 24 (nutrition during pregnancy), the second session was held in weeks 28 and 29 (fetal growth and development, emotional support of fathers during pregnancy, and training on how to become a father); the third session was held in weeks 32 and 33 (planning for childbirth and selecting the type of delivery and analgesia methods), and the fourth session was held in week 37 (infant care). Summary of the contents covered the sessions for each group are reported in Table 2.

**Table 1:** Key points for data collection

**T1**	• Recruit and screen participants at 18–20 weeks • Baseline: A demographic characteristic form
**Allocation**	• Allocation of participants into three groups on a weekly basis: • Study group A: Social media-based training for Spouses in lolagar clinic • Study group B: face-to-face training for spouses in Azadi clinic • Control group: Receiving no education for spouses in Tehransar clinic
**T2**	• Data collection 12 weeks after delivery: MPAS and PPAQ

**Table 2:** The educational content of virtual and face-to-face groups

Time	Content	Objectives	
First session: 24–28 gestational week	Pregnancy diet	- Pregnancy diet with emphasis on what to eat - An introduction to the food pyramid	1 videocast and 2 video files for theoretical content
Second session: 28–30 gestational week	Mental health during pregnancy	- An introduction to the fetal growth and development - Preparing for motherhood - Preparing for fatherhood	2 videocasts, 1 PDF, and 2 video files for theoretical content
Third session: 32–33 gestational week	Planning for delivery and selecting the type of delivery	- Vaginal delivery vs. cesarean section - Different pain control methods during labor - Selecting the delivery location and necessary equipment for delivery	2 videocasts, 1 PDF, and 2 video files for theoretical content
Fourth session: 37 gestational week	Neonatal care	- Neonatal care and risk factors	2 PDF files and 2 videos file for theoretical content

In the virtual group, the spouses received educational content through a Telegram social media App in 4 sessions. In the in-person training group, the spouses received educational contents of the first and third sessions in the form of an educational booklet. The second and fourth sessions of the spouses' training corresponded to the third and eighth sessions of childbirth preparation classes for women. It should be noted that in-person training was conducted by the health center’s midwife through a lecture method for 90 min. The spouses in the control group did not receive any training. All women in three groups participated in childbirth preparation classes. Participants in three groups were followed up for 12 weeks after delivery and the father's attachment to the infant as well as mother’s attachment to the infant were assessed by the Postnatal Paternal-Infant Attachment Questionnaire and Maternal Postnatal Attachment Scale, respectively.

### Participants

Inclusion criteria for women included; being 18–35 years old, having a gestational age of 20 weeks, having the ability to read and write, having low-risk singleton pregnancy, having no history of infertility, and having no history of any physical-mental disorders or smoking, alcohol, and drug addiction by self-report. Inclusion criteria for men included experiencing fatherhood for the first time; having at least 18 years of age; living together with a spouse; ability to read and write; having a cell phone or computer with the ability to install the telegram program until the end of the study; having no history of any physical-mental disorders or addiction to cigarettes, alcohol, and drugs; and having no adverse life events like the loss of loved one in the last 3 months before the intervention.

Withdrawal criteria for men and women included; the occurrence of preterm delivery or any signs of pregnancy risk, hospitalization of the infant in the neonatal intensive care unit, unwillingness to continue the study, not giving feedback for a week to the messages sent to the Telegram group in the virtual education group, and the spouses’ absence in the in-person education group for more than one session.

**Fig. 1:**
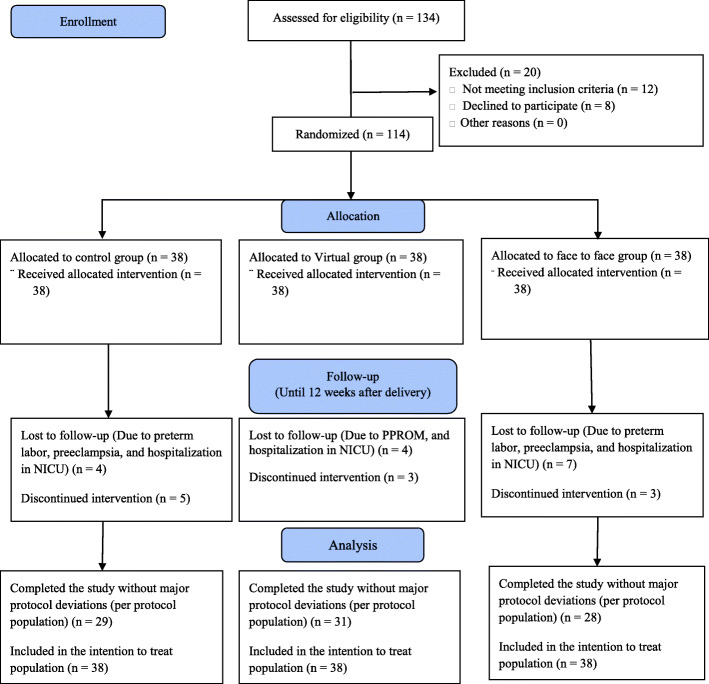
The process of participants’ entry and exit during the clinical trial

In order to collect data, the researcher first selected eligible participants, who attended the prenatal clinics of Lolagar Hospital (virtual education group), Azadi Town Health Center (in-person training group), and Tehransar Health Center (control group), by continuous sampling and provided them with the necessary explanations on the study process. Subsequently, those who gave their consent to take part in the study were enrolled. Recruitment of pregnant women and men by researcher (Z.D) took place over a period of approximately three months. The intervention period began in June 2020. Follow-up ended in May 2021. Figure 1 shows the process of participants’ entry and exit during the clinical trial.

### Assessment of trial variables

The variables in this study were measured as follow:

#### Demographic and midwifery characteristics

A researcher-made questionnaire was designed in three parts. The first part was related to women’s personal characteristics (age, education, economic status, employment, and insurance status), the second part was related to spouses’ personal characteristics (age, education, and occupation), and the third part was related to pregnancy records (gestational age at the time entry to study, number of participated sessions of childbirth preparation classes, body mass index in the first trimester of pregnancy, and pregnancy status (wanted or unwanted)).

#### Measurement of the mother-infant attachment as primary outcome

The Maternal Postnatal Attachment Scale (MPAS) was designed in 1998 by Condon and Corkindale. This tool has 19 items and three subscales including attachment quality (9 items), absence of hostility (5 items), and pleasure in interaction (5 items). The score in this questionnaire ranges from 19 to 95, and the higher scores indicate stronger attachment. The internal consistency of this questionnaire was 0.78 in a study conducted on 200 mothers with 6-month-old infants [28]. In the study of Dezvaree et al., after confirming the validity of the tool, its reliability was determined at 0.9 using the test-retest method [29]. Mothers in three groups were filled up MPAS posted via Telegram social media App 12 weeks after delivery.

#### Measurement of the father-infant attachment as primary outcome

The Postnatal Paternal-Infant Attachment Questionnaire (PPAQ) was developed by Condon et al (2008). This tool has 19 items in three subscales of patience and tolerance (8 items), pleasure in interaction (7 items), and excitement and pride (4 items). The score in this questionnaire ranges from 19 to 95, and the higher scores indicate stronger attachment. In a study conducted on 200 fathers who were experiencing the birth of their first child, the internal consistency of this questionnaire was 0.81 at the sixth month and 0.78 at the twelfth month of childbirth [30]. This questionnaire has been translated into Farsi by Arshadi Bostanabadi et al. The validity of this tool was confirmed after translation. Also, its reliability was confirmed using internal consistency with Cronbach’s alpha of 0.86 [31]. Fathers in three groups were filled up PPAQ posted via Telegram social media App 12 weeks after delivery.

### Sample size

In the current study, the sample size for mother– and father–infant attachment was determined to be 114 individuals according to *α* = 0.05, *β* = 0.2, effect size (ES) = 0.7 [32], and 20% possibility of dropouts (*n* = 38 samples in each of the control, virtual, and face-to-face groups).

$$ n=\frac{2{\left({z}_1+{z}_2\right)}^2}{{\mathrm{ES}}^2}=\frac{2{\left(1.96+0.84\right)}^2}{(0.7)^2}=32 $$

### Ethical consideration

The protocol of the present study was approved by the Ethics Committee of Iran University of Medical Sciences with the ethics code: IR.IUMS.REC.1398.781. It was also registered in the Clinical Trial Registration Center of Thailand (TCTR) with the code: TCTR20200515011 prospectively. All participants were fully informed about the objectives and process of the study and informed consent was obtained from them by the researcher (Z.D). To increase the rate of recruitment, participants were offered a free telephone consultation from the researcher (Z.D), as the sampling was conducted by a midwife. They were also informed that information obtained during the study process will remain confidential, and they can withdraw from the study at any time. No fees were charged for the study and all services were completely free.

### Statistical analysis

The data were analyzed via SPSS software version 22 using descriptive and inferential statistics. Descriptive statistics such as numerical indexes and frequency distribution tables were used to describe the data. One-way analysis of variance (ANOVA) was used to compare the quantitative variables of age, body mass index, number of participation in prenatal classes, and gestational age at the time of entry to study. Fisher's exact test was used to compare the qualitative variables of education level, economic status, employment, pregnancy status, and insurance status. One-way ANOVA was used to compare the scores of mother’s attachment to infant and father’s attachment to the infant. Then, the Tukey post hoc test was used for the two-by-two comparison of the mean scores of father-infant attachment between the groups. The effect sizes in between groups comparisons were reported based on Cohen’s d effect size (null effect = 0, trivial effect = 0–0.19, small effect = 0.2 = 0.49, medium effect = 0.5–0.79, large effect = 0.8–1.19, very large effect = 1.2–2, and huge effect ≥ 2) [33, 34]. It should be noted that the analysis process of the current study was performed by using both intention to treat and per-protocol approaches. Also, the missing mother’s attachment to the infant and father’s attachment to the infant values were imputed with a multiple imputation model [35]. In all tests, a significance level of less than 0.05 was considered.

## Results

### Samples’ characteristics

A total of 134 women were evaluated for their eligibility to participate in the study, from whom 114 eligible women have entered the study. In the in-person group, 10 people were excluded (1 due to preterm labor during the study, 1 due to preeclampsia, 3 withdrew from the study, and 5 were unable to continue due to hospitalization in the newborn intensive care unit (NICU)). In the virtual group, 7 people were excluded (2 withdrew from the study, 1 due to preterm premature rupture of the membranes (PPROM) during the study, and 4 due to Hospitalization in NICU). Also in the control group, 9 people were excluded (1 due to preterm labor during the study, 1 due to preeclampsia, 5 due to unwillingness to continue with the study, and 2 due to hospitalization in (NICU)). The analysis in the current study was performed by both intention to treat and per-protocol approaches on 114 and 88 participants, respectively (Fig. 1). According to the findings, there was no statistically significant difference between the groups in terms of the variables of demographic characteristics and pregnancy records (Table 3).

**Table 3:** Demographics and baseline characteristics of study participants and comparisons between face-to-face, virtual, and control groups

Variables	Face-to-face group (***n*** = 38)	Virtual group (***n*** = 38)	Control group (***n*** = 38)	***P*** value
**Women’s age (year),** mean ± SD	3.17± 27.36	3.74 ± 27.29	5± 27.14	**0.97**
**Husband’s age (year),** mean ± SD	4.12± 32	3.62± 31.35	4.40± 30.66	**0.46**
**Women’s education,***n* (%)				
High school	0 (0)	2 (6.5)	1 (3.4)	**0.87**
Diploma	9 (32.1)	9 (29)	10 (34.5)	
University	19 (67.9)	20 (64.5)	18 (62.1)	
**Husband’s education,***n* (%)				
High school	1 (3.6)	2 (6.5)	1 (3.5)	**0.2**
Diploma	11 (39.3)	19 (61.2)	19 (65.5)	
University	16 (57.1)	10 (32.3)	9 (31)	
**Economic status,***n* (%)				
Good	14 (50)	10 (32.2)	5 (17.2)	**0.1**
Moderate	12 (42.9)	19 (61.3)	20 (69)	
Weak	2 (7.1)	2 (6.5)	4 (13.8)	
**Women’s occupation,***n* (%)				
Housewife	26 (92.9)	25 (80.6)	28 (96.6)	**0.12**
Employed	2 (7.1)	6 (19.4)	1 (3.4)	
**Husband’s occupation,***n* (%)				
Unemployed	0 (0)	1 (3.2)	0 (0)	**0.88**
Worker	1 (3.6)	2 (6.5)	2 (6.9)	
Employed	10 (35.7)	7 (22.6)	8 (27.6)	
Self-employed	17 (60.7)	21 (67.7)	19 (65.5)	
**Insurance status,***n* (%)				
Yes	25 (89.3)	25 (80.6)	25 (86.2)	**0.78**
No	3 (10.7)	6 (19.4)	4 (13.8)	
**Gestational age (at the time of enrollment),** mean ± SD	0.89± 19.14	0.71 ± 19.87	0.79± 19.07	**0.4**
**Number of sessions to attend childbirth preparation classes,** mean ± SD	1.01± 6	1.14 ± 5.87	1.04 ± 5.62	**0.4**
**BMI,** mean ±SD	4.05± 23.80	4.78±25.62	3.58± 24.21	**0.21**
**Pregnancy status,***n* (%)				
Wanted	22 (78.6)	29 (93.5)	23 (79.3)	**0.18**
Unwanted	6 (21.4)	2 (6.5)	6 (20.7)	

^a^*P* < 0.05 is significant, ^b^Standard deviation

### Intervention’s effects on outcomes

As demonstrated in Table 4, there was a statistically significant difference between the three groups in terms of the father-infant attachment score after the educational intervention for both intention-to-treat (*p* = 0.001) and per-protocol (*p* = 0.004) analysis. There was also a statistically significant difference between the two groups of in-person education and control (ITT: 88.37 ± 2.78 vs. 85.19 ± 4.34, *p* = 0.001; PP: 88.14 ± 3.23 vs. 84.66 ± 4.38, *p* = 0.004), and also virtual education and control (ITT: 87.57 ± 3.78 vs. 85.19 ± 4.34, *p* = 0.02; PP: 87.25 ± 4.13 vs. 84.66 ± 4.38, *p* = 0.035). However, no statistically significant difference (ITT: 88.37 ± 2.78 vs. 87.57 ± 3.78, *p* = 0.613; PP: 88.14 ± 3.23 vs. 87.25 ± 4.13, *p* = 0.665) was found between the two groups of in-person and virtual education (Table 4). The results demonstrated a large, medium, and small effect size between the two groups of in-person education and control, virtual education and control, and in-person and virtual education in terms of father-infant attachment score clinically for both intention-to-treat and per-protocol analysis based on Cohen’s *d*, respectively. There was also no statistically significant difference between the three groups after the educational intervention in terms of the mother-infant attachment score for both intention-to-treat (*p* = 0.46) and per-protocol (*p* = 0.64) analysis (Table 5).

**Table 4:** Effect of intervention on outcomes—intention-to-treat sample

**Groups**	**Father-infant attachment**	**Differences between groups**			
	**Mean (SD**^**a**^**)**	**Groups compared**	**MD**^**b**^**(CI**^**c**^**95%)**	**ES**^**d**^**(between groups)**	***P*value**^**e**^**(between groups)**
**Face-to-face group (*n*= 38)**	88.37 (2.78)	F ^**f**^ v/s V ^**g**^	0.79 (-1.2 to 2.79)	0.24	0.613
**Virtual group (*n*= 38)**	87.57 (3.78)	F v/s C ^**h**^	3.18 (1.11 to 5.25)	0.87	0.001
**Control group (*n*= 38)**	85.19 (4.34)	V v/s C	2.38 (0.313 to 4.45)	0.58	0.02
**Groups**	**Mother-infant attachment**	**Differences between groups**			
	**Mean (SD)**	**Groups compared**	**MD (CI 95%)**	**ES (Between)**	***P*value**
**Face-to-face group (*n*= 38)**	85.31 (3.12)	F v/s V	0.08 (-1.89 to 2.05)	0.022	0.46
**Virtual group (*n*= 38)**	85.23 (4.01)	F v/s C	-0.89 (-2.94 to 1.14)	0.26	
**Control group (*n*= 38)**	86.21 (3.63)	V v/s C	-0.97 (-3.02 to 1.064)	0.25	

^a^Standard deviation, ^b^Mean difference, ^c^Confidence interval, ^d^Effect size, ^e^One-way ANOVA with post hoc, ^f^Face-to-face group, ^g^Virtual group, ^h^Control group

**Table 5:** Effect of intervention on outcomes—per protocol sample

**Groups**	**Father-infant attachment**	**Differences between groups**			
	**Mean (SD**^**d**^**)**	**Groups compared**^**e**^	**MD**^**f**^**(CI**^**g**^**95%)**	**ES**^**h**^**(between)**	***P*value (between groups )**
**Face-to-face group**^**a**^**(*n*= 28)**	88.14 (3.23)	F v/s V	0.89 (-1.57 to 3.35)	0.24	0.665
**Virtual group**^**b**^**(*n*= 31)**	87.25 (4.13)	F v/s C	3.48 (0.98 to 5.9)	0.9	0.004
**Control group**^**c**^**(*n*= 29)**	84.66 (4.38)	V v/s C	2.58 (0.14 to 5.03)	0.61	0.035
**Groups**	**Mother-infant attachment**	**Differences between groups**			
	**Mean (SD)**	**Groups compared**	**MD (CI 95%)**	**ES (Between)**	***P*value**
**Face-to-face group (*n*= 28)**	86.13 (3.27)	F v/s V	0.4 (-1.95 to 2.75)	0.11	0.64
**Virtual group (*n*= 31)**	85.73 (4.29)	F v/s C	-0.51 (-2.9 to 1.87)	0.15	
**Control group (*n*= 29)**	86.65 (3.66)	V v/s C	-0.92 (-3.25 to 1.42)	0.23	

^a^Standard deviation, ^b^Mean difference, ^c^Confidence interval, ^d^Effect size, ^e^One-way ANOVA with post hoc, ^f^Face-to-face group, ^g^Virtual group, ^h^Control group

## Discussion

Participants in the three groups of in-person education, virtual education, and control did not show a statistically significant difference in terms of individual variables and pregnancy records at the time of entry to study, and the three groups were homogeneous. Regarding the first objective of this study, the results showed that after the educational intervention, there was no statistically significant difference between the three groups in terms of the mean score of mother-infant attachment. In general, the present study showed that in-person and virtual education of the spouses of primiparous women did not affect on improving the mother’s attachment to the infant.

A study was conducted by Serçekus et al. (2016) on the effect of childbirth preparation classes on mother-infant attachment in Turkey. The 8-week training, which included nutrition during pregnancy and lactation, strategies to overcome the fear of childbirth, coping techniques with labor pain, parents-infant interaction, breastfeeding, and infant care, made no statistically significant difference between the two groups in terms of maternal attachment to the infant. This result is consistent with the finding of the present study [36]. In another study in Singapore, in a mobile-based educational program during pregnancy and postpartum for men, which covered contents such as the process of becoming parents, breastfeeding, mother and infant care, and coping with emotional challenges, the mother’s attachment to the infant was examined. The results of this study showed a significant difference between the two groups in terms of maternal attachment score at one and three months after the delivery [37], as the intervention was able to improve maternal attachment to the infant by providing information to fathers through mobile-based software. This result is not in line with the finding of the present study, which could due to the fact that in the above study part of the training was done after delivery, but in the present study, all training was done during pregnancy.

A study was conducted by Yurtsal and Kocoglu that aimed at examining the effect of a father’s education during pregnancy on mother-infant attachment. Training in the intervention group in this study was done in two ways; using an educational booklet and presenting a video to watch an educational film, and face-to-face training using a question and answer method. The results of this study showed that pregnancy education in fathers led to an increase in maternal attachment to infants. The results of this study are not in line with the findings of the present study in terms of the provision of education to fathers during pregnancy through distance and face-to-face methods and its effect on improving mother-infant attachment [38]. The use of different tools in measuring the degree of maternal attachment to infant can explain the differences in results of these two studies.

Setodeh et al. (2018) in a study, which aimed to examine the effect of fathers’ education during pregnancy on the degree of maternal attachment to infants, reported that the fathers’ education was effective in improving maternal attachment to infants. The educational contents in the above study that were delivered in 4 sessions included familiarity with the concept of attachment and types of attachment skills, ways to control stress and anxiety, familiarity with mental and physical changes during pregnancy, and familiarity with the role of spouse to communicate with the fetus [39]. The differences in the results of the above and present studies can be due to teaching attachment behaviors to fathers, the use of different tools, and different measurement times in the studies.

Awareness of fathers increases the support of the mothers and consequently, increases the understanding of this support by the mothers. This has a positive effect on the mother's mental health and leads to the better adjustment of the mother in the postpartum period, and as a result, it improves the mother’s attachment to the infant [40]. However, in the postpartum period, incompatibility with the mother’s role and additional responsibilities of caring for the infant can negatively affect the mother's attachment to the infant [37]. In the present study, there was no statistically significant difference between the three groups in terms of mother-infant attachment, so that in addition to the two intervention groups, the attachment score was also high in the control group. Obtaining these results in the present study can be due to the fact that all mothers in the present study had participated in childbirth preparation classes. Education provided to mothers during pregnancy, childbirth, and postpartum play an important role in the formation of maternal attachment to infants [41]. The study by Köse et al. showed that prenatal education for mothers leads to better emotional communication with infants and more positive behaviors in the postpartum period [42]. This training, if done in the presence of the father, can increase the effectiveness of educational programs related to mother attachment [19].

Regarding the second objective of this study, the results showed a statistically and clinically significant difference in the father-infant attachment scores of in-person and virtual groups after the allocated intervention compared to the control group. In other words, father-infant attachment scores increased after the intervention in the in-person and virtual groups. Also, the results did not show a statistically and clinically significant difference in the father-infant attachment scores between in-person and virtual groups after intervention. Therefore, the education of spouses of primiparous women by both in-person and virtual methods could improve the father’s attachment to the infant.

Yurtsal and Kocoglu conducted to examine the effect of father’s education during pregnancy on his attachment to the infant. The results of this study showed that education during pregnancy to fathers leads to an increase in the score of father-infant attachment. The results of this study are consistent with the present study [38]. In another study, regarding the effect of childbirth preparation classes on father-infant attachment, the father-infant attachment was assessed 6 months after the delivery using the PPAQ instrument. The results showed no statistically significant difference between the two groups in terms of father-infant attachment score. This study is inconsistent with the present study as the educational intervention in this study had no effect on improving the father’s attachment to the infant [36]. Different results obtained in this study and the present study can be due to differences in the time of measuring the father's attachment to the infant.

Infant care and communication skills training for fathers during pregnancy and postpartum increase fathers’ skills in caring for and responding to infant’s needs and improve parents' mental health [43]. Participation of fathers in childbirth preparation classes and prenatal education is one of the factors that affect fathers’ attachment behaviors [44]. On the other hand, fathers’ anxiety and depression during pregnancy have a negative effect on the father’s attachment to the fetus [45]. Therefore, prenatal education by reducing fathers’ anxiety [46] can have a positive effect on the father’s attachment to fetus and infant.

Since the transition to fatherhood and pregnancy are anxious periods for fathers [47], it is essential to support fathers during pregnancy. Therefore, health care providers are advised to conduct appropriate training programs to support fathers and reduce their anxiety. Since in the present study, virtual education, just like in-person education, improved the father’s attachment to the infant, it is possible to overcome the temporal and spatial barriers to fathers' presence in health programs and in addition to helping fathers, increase the fathers’ support of mothers.

### Strength, limitation, and future research

One of the strengths of the present study is confirming the same effect of in-person and virtual training methods on the father-infant attachment, thus proving that virtual education can also be used to encourage men to participate in childbirth preparation classes. One of the limitations of the present study was the holding of in-person childbirth preparation classes by different trainers, which was beyond our control. However, due to the use of the same educational contents in both groups, this limitation was somewhat controlled. Other limitations of the study included participants potential to obtain pregnancy and childbirth information from other sources during pregnancy, conceivably affecting the accuracy of results. Also, it has not been possible to assign participants randomly. This was because the only center that offered special in-person childbirth preparation education for men was the Azadi Health Center. On the other hand, considering that our study is an educational intervention, there is equally no possibility of blinding. As specialist equipment is required for the delivery of virtual education such as internet access and smartphones, the results of the study may not be generalized to those without such equipment and/or people with less access to the internet. For the special features of the concepts of mother attachment to infant and father attachment to infant, it is not possible to measure it before educational intervention during pregnancy. In the present study, the majority of women wanted to become pregnant. As such, future studies may usefully engage samples of women experiencing unwanted pregnancies to explore whether outcomes may differ in such populations.

## Conclusion and implications for practice

The results of the present study showed that the mean score of father-infant attachment in both in-person and virtual education groups was significantly and clinically higher than the control group. Considering that education by both in-person and virtual methods had the same effect on achieving the same results in the two groups, it is suggested that to increase the participation of spouses of pregnant women in the process of prenatal care, the spouses of pregnant women should have the option to engage in virtual education in addition to in-person training.

## Supplementary Information

**Additional file 1.** CONSORT 2010 checklist of information to include when reporting a randomised trial*.

## Data Availability

No additional data is available due to confidentiality restrictions.

## References

[1] Bowlby J (2008). A secure base: Parent-child attachment and healthy human development: Basic books.

[2] Nuzum D, Meaney S, O’Donoghue K (2017). The spiritual and theological challenges of stillbirth for bereaved parents. J Relig Health.

[3] Ustunsoz A, Guvenc G, Akyuz A, Oflaz F (2010). Comparison of maternal–and paternal–fetal attachment in Turkish couples. Midwifery..

[4] Nelson (2020). Nelson textbook of pediatrics.

[5] Verklan MT, Walden M, Forest S (2020). Core curriculum for neonatal intensive care nursing e-book.

[6] Edwards L. Adaptation to parenthood. Matern Nurs. 1999:449–88.

[7] Yarcheski A, Mahon NE, Yarcheski TJ, Hanks MM, Cannella BL (2009). A meta-analytic study of predictors of maternal-fetal attachment. Int J Nurs Stud.

[8] Bouchard G (2011). The role of psychosocial variables in prenatal attachment: an examination of moderational effects. J Reprod Infant Psychol.

[9] Seimyr L, Sjögren B, Welles-Nyström B, Nissen E (2009). Antenatal maternal depressive mood and parental–fetal attachment at the end of pregnancy. Arch Womens Ment Health.

[10] Murray L, Arteche A, Fearon P, Halligan S, Croudace T, Cooper P (2010). The effects of maternal postnatal depression and child sex on academic performance at age 16 years: a developmental approach. J Child Psychol Psychiatry.

[11] Alhusen JL (2008). A literature update on maternal-fetal attachment. J Obstet Gynecol Neonatal Nurs.

[12] Chang H-C, Yu C-H, Chen S-Y, Chen C-H (2015). The effects of music listening on psychosocial stress and maternal–fetal attachment during pregnancy. Complement Ther Med.

[13] Shoghi M, Sohrabi S, Rasouli M (2018). The effects of massage by mothers on mother-infant attachment. Health Med.

[14] Azogh M, Shakiba M, Navidian A (2018). The effect of cognitive behavioral training on maternal-fetal attachment in subsequent pregnancy following stillbirth. Hayat..

[15] Nematbakhsh F, Kordi M, Sahebi A, Esmaeili H (2007). The effect of mother–infant skin to skin contact on mother’s attachment. Fund Mental Health.

[16] Vakilian K, Khatamidoost F, Khorsandi M (2007). Effect of Kangaroo mother care on maternal attachment behavior before. Hormozgan Med J.

[17] Parsa P, Saiedzadeh N, Roshanaii G, Masoumi SZ (2016). The effect of training on maternal-fetal attachment (MFA) in nulliparous women: A randomized clinical trial. Avicenna J Nurs Midwif Care.

[18] Firouzbakht M, Nikpour M, Salmalian H, Ledari FM, Khafri S (2014). The effect of perinatal education on Iranian mothers’ stress and labor pain. Global J Health Sci.

[19] Sajjadi AS, Zahrakar K, Mohsenzadeh F, Karamnia M, Ym S, Alavinezhad S (2016). Efficacy of maternal fetal attachment techniques on enhancing mother's attachment to the fetus. Dev Psychol.

[20] Tohotoa J, Maycock B, Hauck YL, Dhaliwal S, Howat P, Burns S (2012). Can father inclusive practice reduce paternal postnatal anxiety? A repeated measures cohort study using the hospital anxiety and depression scale. BMC Pregnancy Childbirth.

[21] Cheng CD, Volk AA, Marini ZA (2011). Supporting fathering through infant massage. J Perinat Educ.

[22] May C, Fletcher R (2013). Preparing fathers for the transition to parenthood: Recommendations for the content of antenatal education. Midwifery..

[23] Mortazavi F, Delara M, Akaberi A (2014). Male involvement in prenatal care: impacts on pregnancy and birth outcomes. Nursing And Midwifery Journal.

[24] Mortazavi F, Mirzaii K (2012). Reason of, barriers to, and outcomes of husbands’involvement in prenatal and intrapartum care program based on midwives’experiences: A qualitative study. J Arak Univ Med Sci.

[25] Hamzekhani M, Hamidzadeh A, Vasegh RSF, Montazeri A (2014). Effect of computerized educational program on self-efficacy of pregnant women to cope with childbirth. J Knowledge Health.

[26] Simbar M, Nahidi F, Tehran FR, Ramezankhani A (2010). Fathers’ educational needs for perinatal care in urban iran: A qualitative approach. J Biosoc Sci.

[27] Schulz KF, Altman DG, Moher D (2010). CONSORT 2010 statement: updated guidelines for reporting parallel group randomised trials. Trials..

[28] Condon JT, Corkindale CJ (1998). The assessment of parent-to-infant attachment: development of a self-report questionnaire instrument. J Reprod Infant Psychol.

[29] Dezvaree N, Alaeekarahroudi F, KhanaliAgan L, TalebiGhane E (2016). The mother-newborn s attachment and its related factors in mothers of hospitalized preterm neonates. Health Care.

[30] Condon JT, Corkindale CJ, Boyce P (2008). Assessment of postnatal paternal–infant attachment: development of a questionnaire instrument. J Reprod Infant Psychol.

[31] Bostanabadi MA, Valizadeh S, Rezanezhad J, Jabbari T (2014). Paternal–newborn bonding and its related factors. Nurs Midwifery J.

[32] Craig P, Dieppe P, Macintyre S, Michie S, Nazareth I, Petticrew M. Developing and evaluating complex interventions: the new Medical Research Council guidance. Bmj. 2008;337. 10.1136/bmj.a1655 PMC2769032 18824488

[33] Cohen J (1988). Statistical power analysis Jbr the behavioral.

[34] Sawilowsky SS (2009). New effect size rules of thumb. J Mod Appl Stat Methods.

[35] Tabachnick BG, Fidell LS, Ullman JB (2007). Using multivariate statistics.

[36] Serçekuş P, Başkale H (2016). Effects of antenatal education on fear of childbirth, maternal self-efficacy and parental attachment. Midwifery..

[37] Shorey S, Chee CYI, Ng ED, Lau Y, Dennis C-L, Chan YH (2019). Evaluation of a technology-based peer-support intervention program for preventing postnatal depression (part 1): randomized controlled trial. J Med Internet Res.

[38] Yurtsal ZB, Kocoglu G (2015). The effects of antenatal parental breastfeeding education and counseling on the duration of breastfeeding, and maternal and paternal attachment. Nutr Metab.

[39] Setodeh S, Sharif F, Akbarzadeh M (2018). The impact of paternal attachment training skills on the extent of maternal neonatal attachment in primiparous women: a clinical trial. Fam Med Primary Care Rev.

[40] Khanzadeh Z, Mogaddam TF (2020). The Effect of Supportive Couple-Centered Counselling on Perception of Support for Spouse and Maternal Attachment in Parturients Referring to Health Centers of Urmia University of Medical Sciences in 2018-19. Avicenna J Nurs Midwif Care.

[41] Solt A (2017). Maternal Attachment During Antenatal, Pregnancy and Postpartum Period.

[42] Köse D, Çınar N, Altınkaynak S (2013). Yenidoğanın anne ve baba ile bağlanma süreci. Merhaba..

[43] Nosraty A, Mirzakhani K, Golmakani N, Esmaeili H, Asghari Nekah SM (2019). Effect of Attachment Training on Paternal-fetal Attachment. J Midwifery Womens Health.

[44] Gerner L (2005). Exploring prenatal attachment: factors that facilitate paternal attachment during pregnancy.

[45] Brandão T, Brites R, Pires M, Hipólito J, Nunes O (2019). Anxiety, depression, dyadic adjustment, and attachment to the fetus in pregnancy: Actor–partner interdependence mediation analysis. J Fam Psychol.

[46] Suto M, Takehara K, Yamane Y, Ota E (2017). Effects of prenatal childbirth education for partners of pregnant women on paternal postnatal mental health and couple relationship: a systematic review. J Affect Disord.

[47] Buist A, Morse CA, Durkin S (2003). Men's adjustment to fatherhood: implications for obstetric health care. J Obstet Gynecol Neonatal Nurs.

